# Predictive Structure Emerges During the Generalisation of Kin Terms to New Referents

**DOI:** 10.1162/OPMI.a.27

**Published:** 2025-09-09

**Authors:** Maisy Hallam, Fiona M. Jordan, Simon Kirby, Kenny Smith

**Affiliations:** Centre for Language Evolution, University of Edinburgh, Edinburgh, UK; Department of Anthropology and Archaeology, University of Bristol, Bristol, UK

**Keywords:** kinship terminology, efficient communication, generalisation, transmission biases

## Abstract

Despite cross-linguistic diversity in how kin relations map to terminology, there are constraints on which kin may be categorised together. But what are the constraints on kin term variation, and where do they come from? One proposed constraint is internal co-selection—an evolutionary process where terminological changes in one generation of kin co-occur with parallel changes in other generations. This results in kin categories which are predictable on the basis of other kin categories, a property we call *predictive structure*. To determine the strength of this constraint, we measured the predictive structure of kinship terminology systems from 731 languages. We found that kinship terminologies exhibit a significant degree of predictive structure, and we argue that its prevalence reflects a cognitive pressure for simplicity imposed during the generalisation of known kin categories to new referent types. We tested this claim using an artificial kin term generalisation task. Our results suggest that people do favour predictive structure when generalising from known kin categories to new referents, but that this preference faces interference from other pressures to distinguish kin by features like gender.

## INTRODUCTION

Different languages encode different semantic concepts, as we know from cross-linguistic investigations of semantic domains such as colour (Berlin & Kay, 1969; Regier et al., 2007), number (Greenberg, 1978; Xu et al., 2020), spatial relations (Khetarpal et al., 2009; Levinson & Meira, 2003; Majid et al., 2004), and the focus of this paper, kinship (Greenberg, 1990, 2005; Kemp & Regier, 2012; Murdock, 1949). Consider the English kin term *uncle*, for example. An English speaker can use *uncle* to refer to any of their parents’ male siblings^1^ while a Hindi (Indo-European) speaker has three distinct terms for the same group of kin: *māmā* ‘mother’s brother’, *tāū* ‘father’s older brother’, and *cācā* ‘father’s younger brother’. Meanwhile, in Hawaiian (Austronesian), the term *makuakane* includes all of the male siblings of both parents and also one’s own father. Nevertheless, in spite of surface variation in the way kin are partitioned across different languages, the extent of the possible variation is highly constrained. Some theoretically possible patterns are never attested at all: there is no language, for instance, with a single term meaning only ‘grandfather and brother’, or ‘niece and grandson’. In this paper, we address the extent of these constraints on kinship variation, and propose a mechanism by which they emerge and persist.

### Proposed Constraints on Variation

One means by which we might describe the pattern of constrained variation is through a small set of featural constraints such as gender distinctions, generational distinctions, or relative age distinctions (Fox, 1979; Kroeber, 1909). Perhaps we cannot have ‘niece’ and ‘grandson’ mapped to a single term simply because they differ along dimensions of generation and gender? While tempting, such an explanation cannot capture the scope of the variation in distinctive features we see cross-linguistically. For instance, Omaha (Siouan) categorises kin together from different generations with the term *enä’hä*, meaning ‘father’, ‘father’s brother’, and ‘father’s brother’s son’. In Indonesian (Austronesian), the term *kakak*, meaning ‘older brother’ and ‘older sister’, categorises kin of varying genders together. Furthermore, kinship systems rarely make the same distinctions across the entire paradigm: in English, our grandparents, parents, siblings, children and grandchildren are all distinguished by gender, but not our cousins. More recently, a body of work has situated these featural constraints within a hierarchical framework, creating generative rulesets that can specify the kind of complexity we see in kinship systems cross-linguistically (Greenberg, 1990; Jones, 2010). While rule-based constraints on kin terms are valuable descriptors of variation, these descriptive accounts do not offer an explanation of the mechanisms that give rise to the variation in kinship terminologies that they capture.

Elsewhere in the cognitive sciences, the pattern of constrained variation in kinship systems has been explained using principles of efficient communication (Kemp & Regier, 2012; Kemp et al., 2018). Systems of kinship terminology are constrained by a trade off between opposing pressures to be simple and expressive; systems that fail to trade off these pressures efficiently are unattested. Kinship terminology systems are simpler if they have fewer terms and if these terms have simple, compressible meanings. Disjunctive categories like ‘a sibling who is older than you *or* male’ are dispreferred compared to contiguous categories like ‘a sibling who is older than you *and* male’ (Nerlove & Romney, 1967). On the other hand, kinship terminology systems are more expressive if the probability of correctly interpreting the intended referent of a kin term is maximised. This means that expressive kinship systems have more terms that each denote fewer individuals, and high frequency referents have more precise terms, reducing the chance of a term being misunderstood. This account explains why we have a wide range of surface variation, but also defines clear boundaries on the extent of that variation: for a given level of communicative precision kinship terminology systems are as compressible as possible, and for a given level of compression they are as precise as possible. In short, we shouldn’t expect to find kinship systems that are both complex and highly ambiguous.

Here we seek to understand the scope of diversity in kinship terminology systems from an evolutionary perspective, in tandem with the mechanisms that give rise to that diversity—an under-researched yet rich union between language, culture and cognition (for a review, see Mitchell & Jordan, 2021). Constraints on language variation are not random. They reflect adaptations by languages to their cultural transmission, such that simpler structures that are more learnable, and expressive structures that are more useful for communicating, proliferate (Kirby et al., 2008, 2015; Smith, 2022). We can reformulate the trade-off between simplicity and expressivity in semantic category systems as a joint adaptation to language learning and communication (Carr et al., 2020). Indeed, crosslinguistic constraints on colour terminology have been associated with biases imposed during cultural transmission (Xu et al., 2013), and cultural transmission should similarly be the process by which kinship terminology is optimised for efficient communication (Kemp & Regier, 2012). Previous work offers some insight on the role of learning and communication in the design of kin terms: child language acquisition studies find that semantically simpler kin terms like *father* and *brother*, which require understanding only a single relation, are learned earlier than those like *uncle*, which requires additionally that a child understands both *father* and *brother* (Danziger, 1993; Haviland & Clark, 1974). Experimental work finds that simpler kinship systems (where kin are categorised by shared features) are more learnable by adults in the lab than those where kin terms are shared by less similar individuals (Smith et al., 2020). Additionally, corpus work shows that kinship terms are optimised for the communication needs of individuals, reflecting the relative frequencies with which particular kin are referenced (Anand & Regier, 2023).

### Internal Co-Selection

**Figure 1.**
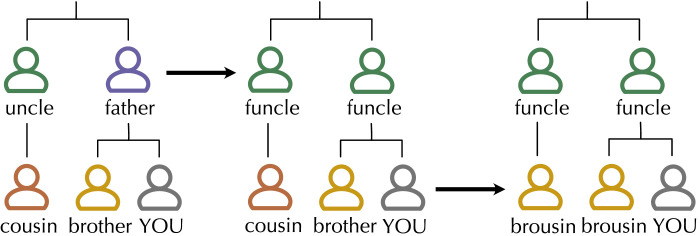
An example of internal co-selection. The system on the left undergoes a merger, such that the previously unique terms *father* and *uncle* collapse to a single term *funcle* which can be used for both referents. Under the internal co-selection hypothesis, we expect a parallel merger in the terms for their children, as it is no longer clear when the child of a *funcle* should be a *brother* or a *cousin* at this intermediate stage. By merging these two terms to a single term *brousin*, we remove this complexity from the kinship system (at the cost of reduced expressivity).

What common features might we then expect systems of kinship terminology to have, if they are constrained by opposing pressures to be simple and expressive? One proposed structural constraint on kinship systems is *internal co-selection* (Passmore et al., 2021): a process of kin term change where new distinctions or mergers in one part of the system are followed by parallel changes in another part of the system. Figure 1 displays an example of this process. If a merger between the meanings ‘father’ and ‘uncle’ results in a single term *funcle* for both referents, we might find ourselves in the confusing situation where sometimes the child of a *funcle* is called our *brother* and sometimes they are called our *cousin*. Choosing the appropriate term for the child of a funcle becomes a difficult task, since we cannot rely on our knowledge of other kin terms to arrive at the correct term. We should therefore expect a second merger between brother and cousin to a single category *brousin*. Then, the child of a *funcle* is always called a *brousin*, and we can rest easy knowing that we do not have to remember complex information about what our *funcle*’s child is called under what particular circumstances.

If we have a single term categorising fathers and uncles, the category structure for their children should not vary arbitrarily, but instead also become constrained to a single term. We refer to this property as the **predictive structure** of the kinship system. The more accurately you can predict the category for an individual in one generation (e.g., sibling and cousin terms) from the category structure for individuals in another generation (e.g., terms for parents, aunts and uncles), the more predictive structure the system has. We propose that predictive structure is one constraint that optimises kinship terminologies for efficient communication, as predictive kinship terminologies are more compressible and therefore simpler: each kin term can be defined with respect to other terms (e.g., we use our understanding of the term *funcle* to define the term *brousin*).

If internal co-selection is a driving force in the evolution of kinship terminology, predictive structure should be common in systems of kinship terminology cross-linguistically. However, Passmore et al. (2021) did not find consistent evidence that kinship systems are structured predictively—at least, not in the ways presumed by common organisational typologies for kinship systems (Murdock, 1949). In Study 1 and Study 2, we extend their study using an alternative, information-theoretic measure of predictive structure in order to more broadly test the prediction that kinship terminology systems are structured predictively. We present the broadest typological analysis of the structure of kinship systems to date, finding that the world’s kinship systems are structured in a way that facilitates greater predictive power between pairs of categories; they are structured more predictively than we would expect if there were no evolutionary pressures imposing this particular kind of structure. By offering a common structural principle that underlies simplicity in kinship systems, we support the claim that kinship systems are optimised for efficient communication.

What then would be the mechanism that facilitates internal co-selection as a process of kin term evolution? Why would kin terms evolve via linked sets of changes? Inspired by explanations for predictive structure in other linguistic domains such morphological paradigms (Ackerman & Malouf, 2013; Johnson et al., 2021), we propose that pressures imposed during cultural transmission incentivise predictive structure. Individuals faced with incomplete input when learning kin terms will predict unknown terms from their existing knowledge: the children of *funcles* sharing a term seems a likely hypothesis, so upon hearing that one *funcle*’s child is a *brousin*, the learner extends that term to children of all *funcles*, regularising the paradigm. In Study 3 we offer an empirical test of whether the generalisation process presupposed by the internal co-selection hypothesis does indeed give rise to predictive structure. We present the results of an artificial language generalisation task, and show that predictive structure emerges when generalising known kin categories to new referent types.

## STUDY 1: HOW COMMON IS PREDICTIVE STRUCTURE?

If kinship terminology evolves by internal co-selection, we would expect that kinship terms in one part of the system will be predictive of other parts of the system. We test this claim by comparing kinship terms across two generations: one’s siblings and cousins (**Ego’s generation** or **G**^**0**^) and one’s parents and their siblings (**Ego’s parents’ generation** or **G**^**+1**^). Previous work has not found consistent evidence that kinship systems exhibit predictive structure (Passmore et al., 2021), but here we conduct a broader test using a larger quantity of data from a greater variety of languages, and using a richer measure of predictive structure.

Passmore et al. (2021) measure predictive structure using a binary measure inferred from the typology for kinship systems put forward by Murdock (1949), which classifies kinship systems into six categories depending on the way a language distributes kin terms for cousins. In their study, if the organisation of kin at G^+1^ is classified the same as kin at G^0^, then that kinship system scores 1 (predictive structure). If the two categories differ, the language scores 0 (no predictive structure). However, this measure assumes that the ideal state for kinship systems following the internal co-selection process is one of Murdock’s six types, which do not necessarily exhibit predictive structure as we have defined it here. Kinship systems can be predictive in ways that are not captured by these six types; in particular, a system might be partially predictive, such that some pairs of terms in the system might be predictive of each other, but not others. Additionally, a binary measure cannot capture the extent to which a system exhibits predictive structure given other constraints, such as the size of the kinship lexicon or what other distinctions are made in the system.

Furthermore, while Murdock’s typology is still commonly used in kinship research as a measure of kin term variation (Cronk et al., 2019; Trautmann & Whiteley, 2012) or to infer social kin organisation (Schulz et al., 2019), it is increasingly perceived as an inadequate and outdated description of global kinship variation (Passmore & Jordan, 2020). Indeed, Passmore et al. (2021) suggest that their inconclusive result reflects the fact that the typology fails to accurately capture the extent of global variation.

Here, we use a new information-theoretic measure for predictive structure. We calculate the symmetric conditional entropy of the terms in G^0^ and G^+1^, and use Monte Carlo methods to compare each kinship system to a null baseline, measuring the extent to which systems exhibit predictive structure relative to the amount of predictive structure we would expect to occur by chance in a system with the same number and distribution of terms.

### Data

Our data on the world’s kinship systems comes from Kinbank (Passmore et al., 2023), a database of kinship terminology in 1215 languages across 21 major language families. Kinbank contains terms for relatives spanning five generations from grandchildren to grandparents. For our analysis, we look only at kin terms for individuals in G^0^ including siblings (elder and younger) and parents’ siblings’ children, and in G^+1^ including parents and parents’ siblings (elder and younger). Taking into account gender, this leaves us with a total of 60 kin types across both generations. See Appendix A for a detailed description of the kin types we used and how we processed the data.

Limiting our analysis to two generations aligns with Passmore et al.’s (2021) claim about internal co-selection, which only considers the evolutionary link between G^0^ and G^+1^ (though there may be co-evolutionary links between other kin; Sheard et al. (2020) identify a process akin to co-selection between grandparent and grandchild terms). Additionally, these kin relations are the most reliably documented across the dataset, which allows us to maximise the size of our data sample. When data was missing, we calculated the predictive structure of the terminology that was available, providing there was at least one term listed for a person in each generation. This criterion left us with data from 731 kinship systems for our analysis.

### Measuring Predictive Structure

For our analysis, we need a robust measure of predictive structure and a baseline model against which to compare natural languages. The baseline should capture our expectations under the null hypothesis; i.e., that if there were no evolutionary pressure for predictive structure, kinship terminologies would not be structured in this manner.

#### Symmetric Conditional Entropy.

We measured the predictive structure between two generations of a kinship system as the sum of the conditional entropies of each generation of kin terms given the other, a measure we refer to as **symmetric conditional entropy**. We calculated this measure for each language in our dataset (see Appendix B for a worked example).

To calculate the conditional entropy of one generation of kinship terminology given another, we measured the conditional entropy over pairs of kin categories. Each pair of kin categories consists of a term for an individual in G^+1^ (*T*_1_) and a term for their child in G^0^ (*T*_0_); e.g., *uncle* and *cousin* in English. The conditional entropy of G^0^ given G^+1^ is given by:

$$H\left(G^{0}|G^{+1}\right)=-\sum_{\begin{array}{c} T_{0}\in G^{0},\\ T_{1}\in G^{+1} \end{array}}p\left(T_{0},T_{1}\right)\;{\text{log}}_{2}\frac{p\left(T_{0},T_{1}\right)}{p\left(T_{1}\right)}$$ (1)

where *p*(*T*_0_, *T*_1_) is the probability that *T*_0_ is the label for the child of *T*_1_ and the sum is over all pairs (*T*_0_, *T*_1_) where *T*_0_ and *T*_1_ are a parent-child pair. For a pair of terms (*T*_0_, *T*_1_), the conditional entropy of *T*_0_ given *T*_1_ tells us how many bits of information we need to accurately predict the label *T*_0_ for the child of an individual labelled *T*_1_ given that we already know which individuals are labelled *T*_1_.

The symmetric conditional entropy *H*_*sym*_ is the sum of the conditional entropy of each generation given the other:

$$H_{\mathrm{sym}}\left(G^{0};G^{+1}\right)=H\left(G^{0}|G^{+1}\right)+H\left(G^{+1}|G^{0}\right)$$ (2)

resulting in a value that tells us the variation in information between the two generations: how much extra information we would need to fully predict the category structure at G^0^ given than we know the category structure at G^+1^ and vice-versa.

**Figure 2.**
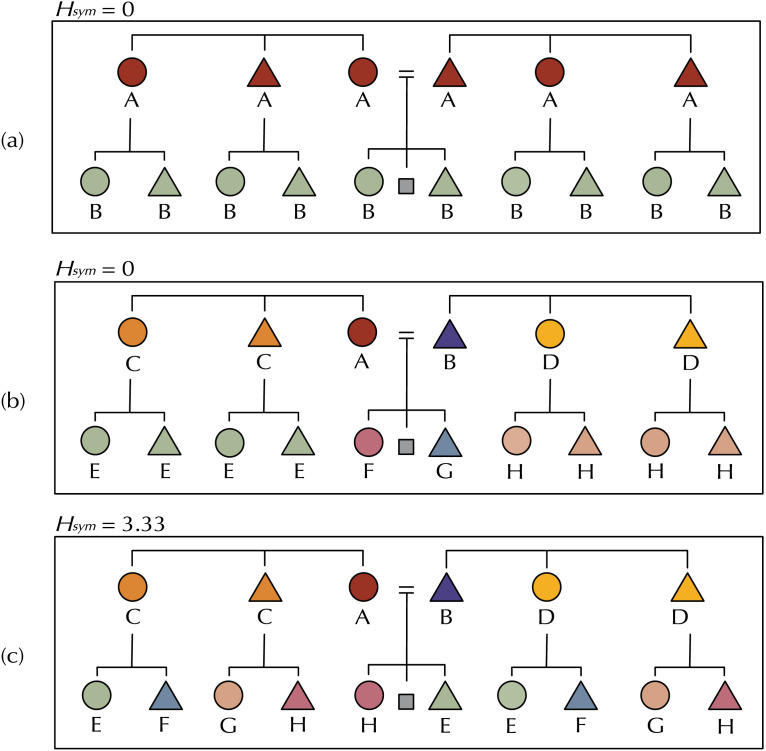
Hypothetical kinship systems exemplifying high and low predictive structure with varying numbers of kin terms. All terms are given relative to Ego, represented by the grey square. Circles represent women, triangles represent men. Colours indicate a unique kin term. Although they vary in number of terms, kinship systems (a) and (b) both exhibit *H*_*sym*_ = 0. Each term in G^+1^ is predictable from a term in G^0^ and vice-versa: in (b), the child of A is always E, the child of B is always F, the child of D is always H, et cetera. System (c) has *H*_*sym*_ = 3.33, low predictive structure, despite also having eight terms. In (c), there is no reliable way to predict a term in G^+1^ from any term in G^0^ or vice-versa: the child of C could be E, F, G or H; and the parent of H could be A, B, C or D.

**Figure 3.**
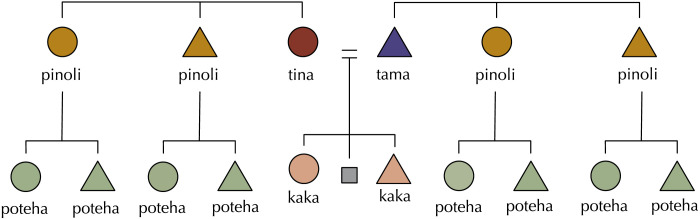
The kinship system of Tolaki (Austronesian). Tolaki has a high degree of predictive structure: the term for cousins *poteha* are uniquely specified by the term for parents’ siblings *pinoli*, the terms for parents *tina* and *tama* uniquely pair with the term for siblings *kaka*. Note: Tolaki does not achieve *H*_*sym*_ = 0, as the distinction between *tina* and *tama* adds uncertainty when predicting the label for the parent of a *kaka*.

**Figure 4.**
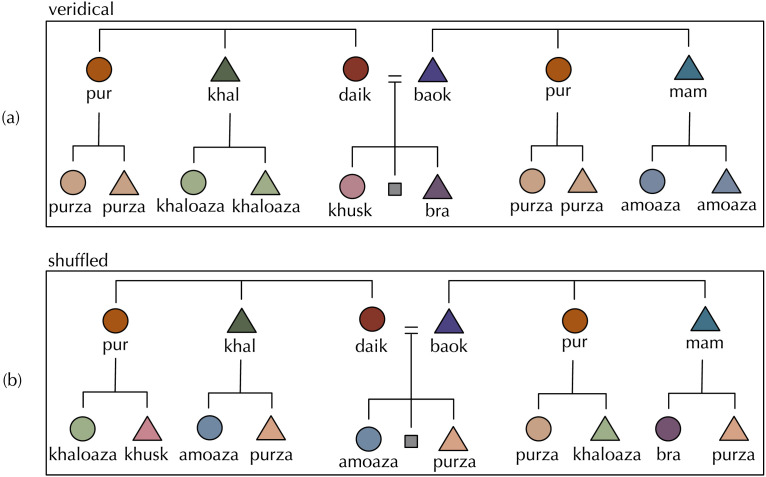
(a) The kinship system of Kurdish (Indo-European). Tree conventions as in Figure 2. Kurdish has a high degree of predictive structure, but these kin terms are also compositional: e.g., *pur* and *purza* share part of their form. (b) An example permutation of Kurdish. This hypothetical kinship system has low predictive structure: there is no reliable way to predict what a child will be called on the basis of what their parent is called. Before, the child of a *pur* was a *purza*; now the child of a *pur* might be *amoaza*, *bra*, *purza* or *khusk*.

*H*_*sym*_(*G*^0^; *G*^+1^) = 0 when every term in G^0^ is uniquely specified by a term in G^+1^ and vice-versa, and is maximised when there is no reliable way to predict with certainty any term at G^0^ given a term in G^+1^ and vice-versa (example kinship systems are given in Figure 2). In reality, most languages sit between these extremes, like Tolaki (Austronesian; Figure 3) or Kurdish (Indo-European; Figure 4a), but we predict that for a given amount of variation kinship systems have lower symmetric conditional entropy between generations than we would expect if kin terms were distributed across the space of possible referents in the absence of any pressure for predictive structure.

#### A Baseline With No Bias for Predictive Structure.

We used Monte Carlo techniques to test whether a given level of predictive structure is likely to arise by chance, comparing each language’s true symmetric conditional entropy score to a null baseline with no bias for predictive structure, but with all other features of the language held constant. For each language, we generated baseline kinship systems by generating random permutations of the kin terms in G^0^, randomising which kin terms were associated with which referents, and thereby breaking any predictive bonds between terms in G^0^ and terms in G^+1^ while preserving the number of terms in each generation. Figure 4b exemplifies this manipulation for the Kurdish kinship system.

For each of the 731 natural kinship systems in our dataset, we generated 1,000 such permutations and measured the symmetric conditional entropy of each permutation. We compared the resulting distribution of theoretically possible symmetric conditional entropy values to the symmetric conditional entropy of the veridical kinship system. We calculated a *z*-score for each language using the mean and standard deviation of the baseline distribution: how many standard deviations the veridical symmetric conditional entropy is from the baseline mean. This indicates how extreme the observed predictive structure is relative to what would be expected if there were no bias for predictive structure. We classified a kinship system as having a significant degree of predictive structure if it had lower symmetric conditional entropy than 95% of its permutations (i.e., a significance threshold of *p* = 0.05), measured as a symmetric conditional entropy value more than 1.96 standard deviations below the baseline mean (*z* < −1.96).

#### Capturing Intuitions About Predictive Structure.

Symmetric conditional entropy captures our intuitions about predictive structure in two key ways. Firstly, it captures our intuition that the ideal kinship system under a pressure for predictive structure is one where we can perfectly predict the labels for categories in either generation. In this case, *H*_*sym*_(*G*^0^; *G*^+1^) = 0. Additionally, our measure captures that kinship systems trade off the pressure for predictive structure—a pressure for simplicity—against a pressure be informative, as it measures the extent to which a kinship system is structured predictively *with respect to* the other distinctions it encodes—i.e., how well it balances simplicity of structure with precise expression.

Secondly, symmetric conditional entropy captures the intuition that predictive structure is symmetric. In the example in Figure 1, if we guessed at random whether the child of a *funcle* was called a *cousin* or *brother*, we would be right 50% of the time. On the other hand, knowing the term *cousin*, we can be 100% certain about the category at G^+1^—the parent of a *cousin* is always called a *funcle*. The *brousin* merger makes the meaning of *brousin* as informative about the meaning of *funcle* as *funcle* is informative of *brousin*. Symmetric conditional entropy captures our intuition that the relationship between *funcles* and *brousins* should be similarly predictive in both directions (and if it is not, then the system will have a lower symmetric conditional entropy). Predictive structure has been measured as standard conditional entropy elsewhere in the literature (Ackerman & Malouf, 2013), but by making the measure symmetric, we avoid encoding in our measure any assumptions about the order in which kin terms are learned, or about the direction of prediction.

### Results

Our hypothesis is that systems of kinship terminology exhibit an extreme degree of predictive structure, following the prediction from Passmore et al. (2021) that kin terms evolve via a linked set of changes. In terms of our measure, we expected that natural languages will have a lower symmetric conditional entropy than 95% of their permutations.

**Figure 5.**
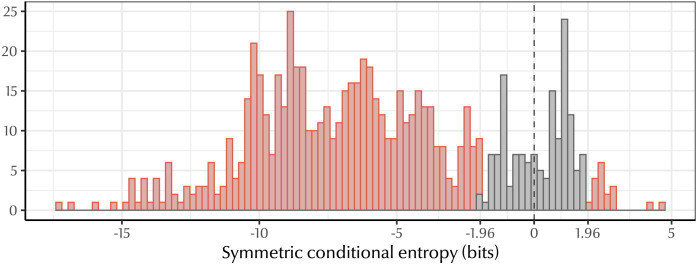
Distribution of *z*-scores for all languages in our dataset. Since predictive structure is associated with lower symmetric conditional entropy, *negative z-scores* indicate greater predictive structure. Red bars indicate languages with significant symmetric conditional entropy scores (*p* < 0.05). Dashed line shows *z* = 0. The majority of languages exhibit a significant degree of predictive structure, and many beat their baseline by an extremely large margin of >5 standard deviations below the mean.

Comparing each natural language to its bespoke baseline, we found that the majority of languages in our dataset (77%) had significantly lower symmetric conditional entropy than their null baselines (see Figure 5). Only 2.6% of languages had symmetric conditional entropy values significantly higher than their baseline; the remaining 20.4% of languages had values in the range we would expect by chance if there were no bias for predictive structure (−1.96 < *z* < 1.96).

**Figure 6.**
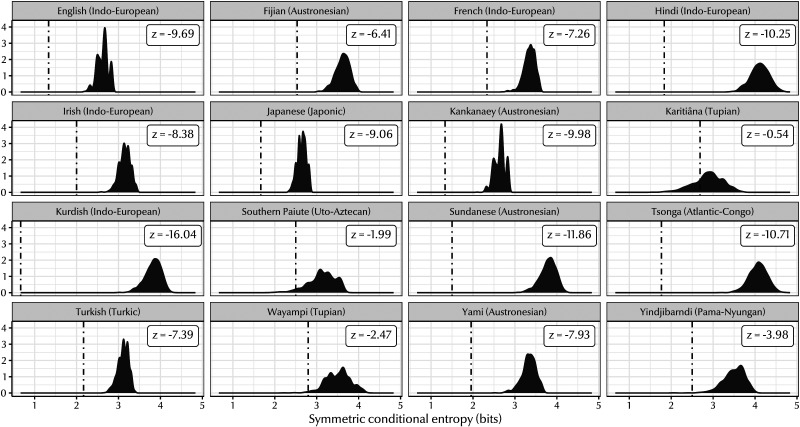
Symmetric conditional entropy values for a sample of languages in our dataset. Distributions show the range of values produced by 1,000 permutations of each system; dotted lines represent the symmetric conditional entropy of the veridical kinship system. Labels give the *z*-score for each language; *z* less than 1.96 indicates a significant degree of predictive structure. In almost all cases, the true symmetric conditional entropy is far less than the bulk of the baseline distribution, indicating that systems of kinship terminology are structured more predictively than expected if there was no bias for predictive structure.

Figure 6 shows the results for a sample of languages in our dataset. For almost all of these languages, the veridical symmetric conditional entropy was significantly lower than the baseline distribution. This was often by a considerable margin, with many languages having veridical values more than five standard deviations below their baseline mean.

### Discussion

In accordance with our hypothesis, we found that kinship systems have much greater predictive structure than we would expect if there were no bias for predictive structure; there is often not a single permutation out of 1,000 that approaches the true symmetric conditional entropy value. This confirms quantitatively that kinship terminologies are structured predictively, and that this is a pervasive property across languages.

We found some languages whose symmetric conditional entropy was not significantly different from their permuted counterparts: shuffling which terms denote which meanings did not make them more or less predictive. This could be for two reasons. First, if a system had only a small number of terms, then the shuffling manipulation would have fewer possible outcomes; a language with only two terms has far fewer distinct permutations than a language with 50 terms, meaning that it would be more difficult for the veridical kinship system to beat its baseline. For example, the kinship system in Figure 2a has only one term in each generation. Although it has *H*_*sym*_ = 0, all possible permutations of this system also have *H*_*sym*_ = 0. As a result, the z-score for this system is also 0; it does not have more predictive structure than its average permutation.

A second reason a kinship system may not show a significant degree of predictive structure is if it is organised only partially predictively, such that shuffling could produce systems both more and less predictive than the original. Why might a system only have partially predictive structure? As we will see in the next section, there are other constraints on kinship systems that might pull them away from maximum predictive structure; for instance, languages might achieve compressibility through compositional structure at the morphological level rather than through predictive structure at the system level.

The strength of our results contrasts with the findings from Passmore et al. (2021), who did not find consistent evidence that kinship systems evolve by internal co-selection. By measuring predictive structure as the symmetric conditional entropy between two generations of kin terms, we were able to show the extent to which a particular kinship system exhibits predictive structure, while Passmore et al.’s (2021) measure was binary—either kin terms were predictive or they were not. By comparing our results to a null distribution, we were able to measure for each language how extreme the degree of predictive structure was. Additionally, we used a larger quantity of data from a greater variety of languages. We are confident that the results we have presented here are a stronger test of predictive structure.

## STUDY 2: IS PREDICTIVE STRUCTURE DUE TO COMPOSITIONALITY?

An alternative explanation for our findings in Study 1 could be that predictive structure is a by-product of compositionality, the pervasive feature of languages whereby the meaning of an expression is composed of the meanings of parts of that expression. It is not uncommon for kin terms to be compositional: morphologically transparent such that parts of their form are associated with parts of their meaning. For instance, Kurdish *khal* ‘father’s brother’ and *khaloaza* ‘father’s brother’s child’ share part of their form, such that *khal* indicates which branch of the family tree these kin belong to; i.e., the father’s brother’s branch (see Figure 4a). Additionally, the suffix *-aza* indicates removal by one descending generation (Barth, 1953), and is also present in the other cousin terms *pur-za* and *am-oaza*. Labels which are compositional in this way inevitably have a high degree of predictive structure.

Compositionality has itself been argued to be in part a product of pressures for efficient communication (Kirby et al., 2015). Could the predictive structure we see in Study 1 be due entirely to compositionality? If so, it would no longer be clear that predictive structure emerges due to a specific pressure for kin categories to uniquely specify one another, but could instead emerge as a side effect of a more general preference for compositional structure in language.

Many of the kinship systems we see that have low symmetric conditional entropy relative to their baselines are not compositional in the same way as Kurdish—e.g., Tolaki in Figure 3, and of those in Figure 6, English, Fijian, Turkish, Kankanyaey, Yami, and Yugambeh have no consistent compositional structure between pairs of parent and child kin terms. But in order to more rigorously rule out the possibility that low symmetric conditional entropy is merely compositionality in disguise, in this section we measure the compositionality of terms denoting a parent and child pair (e.g., *uncle* and *cousin* in English) across our sample of kinship systems, and compare this to the measure of predictive structure for each language as computed in Study 1.

### Data

The data used in this analysis are identical to those described in Study 1.

### Measuring Compositionality

As a proxy for compositional structure between parent and child kin terms, we measured the normalised Levenshtein distance (Levenshtein, 1965) between these pairs of terms^2^: the number of single-letter insertions, deletions, or replacements needed to turn one string into another, proportional to the length of the longer string. For instance, the number of changes required to change the term *uncle* into the term *cousin* is 6, as none of the letters are in the same position and one letter must be added. The length of the longer word *cousin* is 6, so the normalised edit distance between these terms is 1, the highest possible edit distance between a pair of words. To take an example from a language that does exhibit compositionality, consider Kurdish *pur* ‘mother’s sister’ compared to *purza* ‘mother’s sister’s daughter’—the normalized edit distance between these terms is 0.4. The smaller the normalised edit distance, the more similar the two strings; a distance of 0 indicates that the strings are identical. In a kinship system that is compositional in the same way as Kurdish, with terms for offspring being similar in form to terms for their parents, the edit distance between parent and child terms will tend to be low.

We computed the average edit distance between all pairs of parent and child terms. As in Study 1, we built null baselines for each kinship system by permuting which G^0^ terms were associated with which referents and measured the new average edit distance across the new set of parent-child pairs generated by this process. We calculated the z-score of the veridical edit distance with respect to this random baseline. Kinship systems were considered significantly compositional if the true edit distance was smaller than 95% of its permutations (*z* < −1.96), i.e., there is a greater-than-chance resemblance between the form of the labels for parents and their children.

### Results

**Figure 7.**
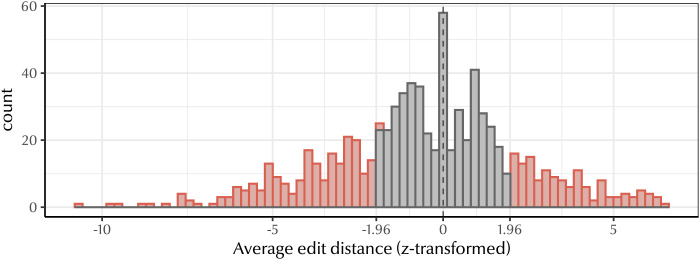
Distribution of average edit distance z-scores for all languages in our dataset. Since compositional systems have lower edit distances, *negative z-scores* indicate greater compositionality (the average distance between terms in the system is *less* than chance). Red bars indicate languages with significant average edit distance scores (*p* < 0.05); grey bars indicate languages which do not have significant average edit distance. Dotted line shows *z* = 0. Only a small proportion of languages are compositional (26.5%), while predictive structure is more common (77%), suggesting that it is possible to have a high degree of predictive structure without compositionality in kin term forms.

Figure 7 shows the average edit distance z-score for each natural language. We found that languages tend not to have a significant degree of compositionality with respect to their null baselines. 73.5% of languages either do not differ from the baseline or have significantly high edit distance (i.e., are non-compositional), while only 26.5% of languages have a significantly low average edit distance (i.e., are highly compositional). Given that extreme values of predictive structure are much more common (77%), this suggests that predictive structure is not merely an artifact of compositionality.

**Figure 8.**
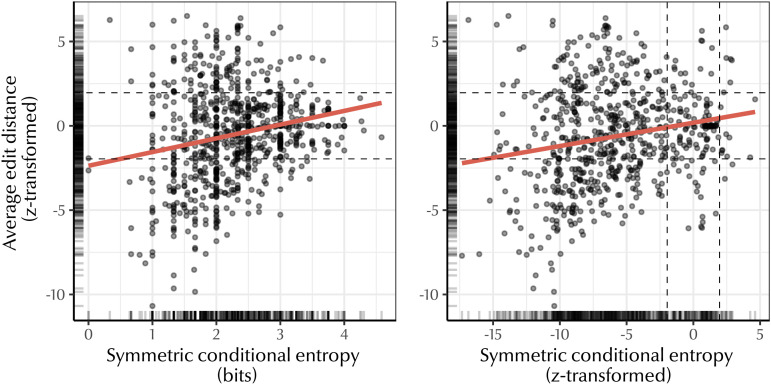
Left: Relationship between z-transformed average normalised edit distance and symmetric conditional entropy. There is a small effect: as edit distance decreases (i.e., terms are more similar), so does symmetric conditional entropy (terms are more predictive). Right: Relationship between z-transformed average normalised edit distance and z-transformed symmetric conditional entropy. Dashed lines indicate significance thresholds. Many languages have significantly low symmetric conditional entropy but non-significant or significantly high average edit distance, suggesting that predictive structure is not driven solely by compositionality.

To confirm that predictive structure is not wholly conditional on compositionality, we compared the z-transformed average edit distance between parent-child terms with the symmetric conditional entropy score for each language. Figure 8 depicts the relationship between these variables: as kin terms become more compositional, predictive structure increases, as we expected. More crucially, we found that 56.6% of kinship systems did not differ significantly from their baseline edit distance. Of this 56.6%, 61.1% still had a significant degree of predictive structure, suggesting that predictive structure need not co-occur with compositionality.

**Figure 9.**
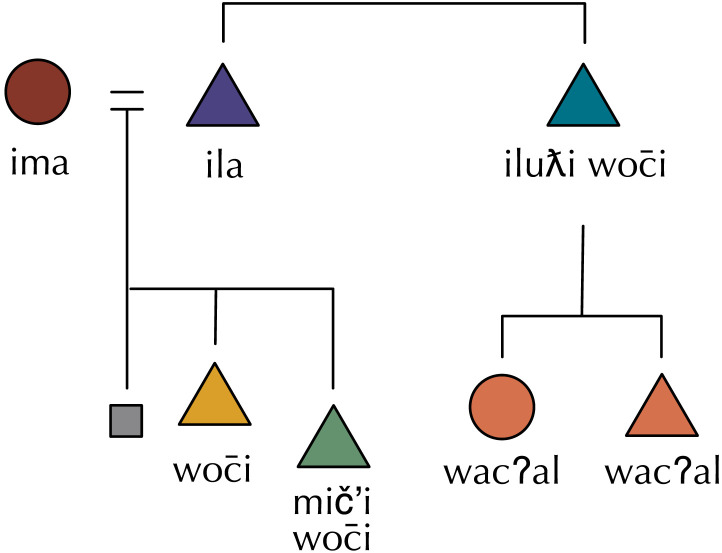
Truncated kinship system for Andi (Nakh-Daghestanian) including terms for parents, male siblings (elder and younger), father’s brother, and father’s brother’s children. Tree conventions as in Figure 2. Longer lines indicate younger siblings, shorter lines indicate older siblings. Andi has extremely high average edit distance according to our measure because terms that share parts of their form are not parent-child pairs: *iluƛi woc̄i* ‘father’s brother’ shares a chunk of its form with *woc̄i* ‘elder brother’ compared to *wacɁal* ‘cousin’.

Additionally, we also found that 16.9% of kinship systems have edit distances that are significantly *greater* than those in the baseline, meaning that the labels for corresponding pairs of G^+1^ and G^0^ kin (i.e., parents and their children) are less similar to each other than they are to other kinship terms in the system. Our edit distance measure is highly specific to the cross-generational predictive structure we identified in the Introduction, only measuring the wordform similarity between terms for a parent and their offspring. However, this is not the only manner in which languages encode meaningful information in kin terms. For instance, Figure 9 shows the kinship system for Andi, where the kin terms for ‘brother’ and ‘father’s brother’ share a chunk of their form, -*woc̄i*. This morpheme encodes a meaning like ‘this person is a male sibling’. Our edit distance measure would give a higher score to Andi if the term *woc̄i* was used for ‘father’s brother’s son’—any permutation that pairs these two terms as a parent-child pair will have a lower average edit distance.

Importantly, the majority of systems with significantly high edit distance values (i.e., less compositional than chance, according to our measure) nevertheless still have significantly low symmetric conditional entropy values (85.6%). For instance, Andi has a edit distance *z*-score of 6.4, and a symmetric conditional entropy *z*-score of −9.34 (*p* < 0.001), further supporting our claim that predictive structure is not merely a reflection of compositionality.

### Discussion

While predictive structure and compositionality are intrinsically related, our results suggest that predictive structure is not contingent on kin terms being compositional and non-compositional kin terms can exhibit predictive structure. Because extremely high edit distance values can correspond with extremely low symmetric conditional entropy values, we suggest that not only do predictive structure and compositionality not need to co-occur, but also that they may interact to impose multiple kinds of structure on a kinship system simultaneously: predictive structure allows us to predict who is the descendant of whom, while compositionality encodes information about the shared features of individuals.

## STUDY 3: DOES PREDICTIVE STRUCTURE EMERGE DURING GENERALISATION?

What mechanisms give rise to predictive structure in kinship terminology? Cross-linguistic universals are evidence that language reaches “stable engineering solutions” that satisfy the demands of the processes of language learning and use by which languages are transmitted (Evans & Levinson, 2009). Indeed, typologically common structural properties of language such as noun-dependency ordering (Culbertson, Schouwstra, & Kirby, 2020; Culbertson et al., 2012), harmonic ordering of phrasal heads (Culbertson, Franck, et al., 2020), and conditioned variation more generally (Hudson Kam & Newport, 2009; Smith & Wonnacott, 2010) have been shown to emerge by the imposition of cognitive biases associated with language learning. We argue that the prevalence of predictive structure in kinship terminology suggests a pressure for this structure, and that this pressure is also imposed during learning.

We draw a parallel with the predictive structure of morphological paradigms (e.g., the organisation of number, person or case inflections within a language), which vary in their complexity along two dimensions: the number of different surface forms in the paradigm, and the predictive structure of the paradigm—how reliably you can predict a form from other forms in the paradigm (Ackerman & Malouf, 2013; Johnson et al., 2021). Ackerman and Malouf (2013) note that while variability in surface forms seems to be quite unconstrained cross-linguistically, predictive structure seems to be much more constrained—even morphological paradigms with a great deal of superficial complexity are structured predictively, just as we find with paradigms of kinship terminology. Experimental work has shown that predictive structure licenses reliable generalisations about novel forms when learning morphology (Johnson, 2021); people are more likely to correctly infer the form for an unknown cell if it can be predicted using knowledge from another part of the paradigm.

There are two kinds of generalisation we can make with our kin term knowledge. The first—and the one we address in our experiment—occurs during the learning process. In this scenario, we have knowledge (or partial knowledge) of some of the kin categories in our language, but not all of them. We use what we know to infer what we do not. For instance, we may know that *funcle* is the label for the category containing our male parent and also the male sibling of our parent, but we may not know the category structure that applies to these individuals’ children. Our expectation is that a learner would be more likely to generalise from their knowledge of the category *funcle* to infer that all children of *funcles* should be categorised together (as *brousins*), rather than to infer that the category structure is different for the children (i.e., to infer that sometimes the child of a *funcle* is a *cousin* and sometimes a *brother*). Crucially, we can also infer who should *not* go in the same category—i.e., if two kin in G^+1^ have distinct terms, we can infer their children should not be categorised together.

The second kind of generalisation involving kin terms is applying a known system of kin terms to new individuals in the world. When we meet a relative we haven’t met before, we determine which category they fit in best based on our knowledge of the categories other members of our family belong to. For example, if we meet an individual who fits in the category *brousin*, then we can infer that their father is our *funcle* due to predictive structure. In this scenario, we know the meanings of all kin terms in our language, and we are inferring how new tokens should be categorised into those types.

Both of these processes benefit from predictive structure, though they may benefit differently; for example, a kinship system with a unique term for each relative in G^0^ and G^+1^ is still highly predictive but is nonetheless prohibitively difficult to learn (though perhaps aids learning insofar that we could infer that no two individuals share a term). On the other hand, once we’ve managed to acquire such a system, its predictive structure comes in handy when applying kin terms to new individuals. Nevertheless, here we argue that a bias for predictive structure in the categorisation of meanings (*not* specific individuals) is critical in the emergence of kinship terminology systems with predictable categories: kin terms evolve to suit the kinds of generalisations we are likely to make about category membership during the process of kin term acquisition.

To test whether predictive structure emerges during generalisation, we ran an artificial language experiment where participants generalised known kin terms to novel referents, taking inspiration from similar experiments where participants learn to generalise novel words in a hierarchical meaning space (e.g., Xu & Tenenbaum, 2007). Participants were shown family trees with some members labelled with a kin term and some left unlabelled. Their task was to assign a kin term to each of the unlabelled referents. This paradigm let us test whether participants have a preference for predictive structure when generalising kin terms.

### Methods

#### Participants.

We recruited 40 participants from Prolific. Participants were self-reported native speakers of English resident either in the United Kingdom or the United States who had an approval rating of over 98% over all completed studies. We excluded three participants who failed at least two of eight attention checks, leaving 37 participants. The median completion time for the experiment was approximately 16 minutes, and participants were paid £2.86 for their participation.

#### Design.

We provided participants with incomplete sets of kin terms in artificial languages. Framed as a language documentation exercise, their task was to generalise from these known terms what the remaining terms should be—specifically, we asked them to use the distinctions made amongst terms for parents and their siblings to infer distinctions amongst siblings and cousins.

**Figure 10.**
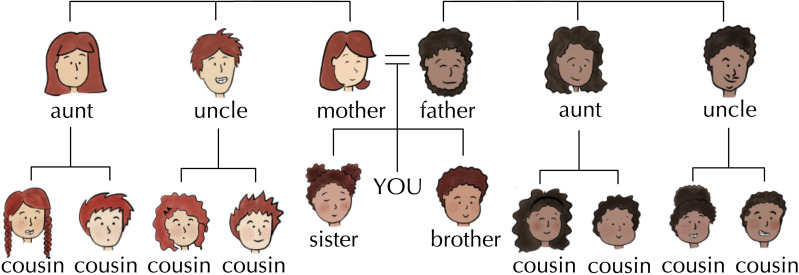
Example family tree diagram from our experiment, labelled here with English kin terms for clarity. Each tree comprised two generations of kin, including a mother, father, maternal/paternal aunt, maternal/paternal uncle in G^+1^ and a male sibling, female sibling, and a male and female child for each aunt and uncle in G^0^. Participants saw a new tree with new characters in each of their eight trials.

We presented participants with a family tree diagram comprising two generations of kin (see Figure 10). The family trees were populated with illustrated characters designed to share visual similarities; closely related kin shared features such as hair colour, hair style, and skin tone. All family trees were oriented with the parents and parents’ siblings at the top with their children positioned beneath them. Participants were encouraged to imagine themselves at the centre of the tree; the word ‘YOU’ was written beneath the parents and between the siblings to indicate how they should imagine themselves to be related to everyone else in the tree. To avoid effects associated with the position of different relatives on screen, we varied the left-to-right order of men and women in the tree between participants, as well as whether the mother and father appeared on the right- or the left-hand side.

**Figure 11.**
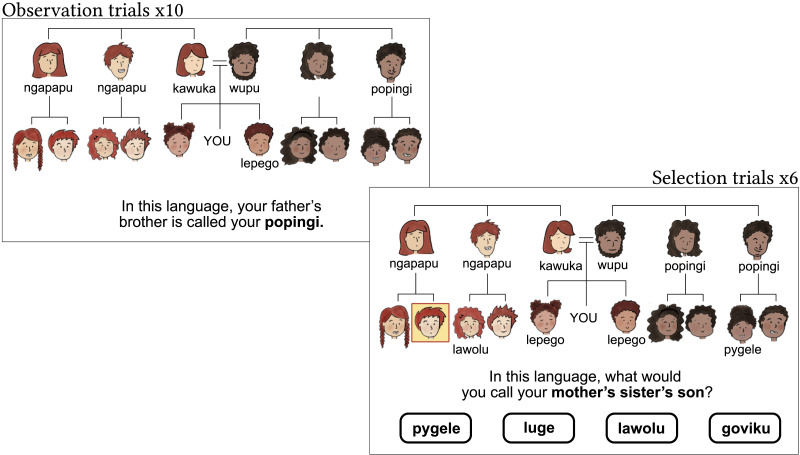
Left: Example observation trial for ‘father’s brother’. Participants saw each of the 10 known kin terms appear in the tree with a prompt telling them the term for that relative in an artificial language. Right: Example selection trial for ‘mother’s sister’s son’. Kin who were labelled in the observation trial remained labelled. One by one, the six remaining kin were highlighted and participants were prompted to choose the label that best fit that referent. Once all kin were labelled, participants could choose to move on to the next trial.

Using this tree diagram, we showed participants an incomplete artificial system of kinship terminology; terms were displayed beneath the corresponding characters in the tree (see Figure 11). We gave participants all labels for G^+1^ kin, and also gave them at least two labels in G^0^ so they could generalise these labels to the remaining unlabelled kin. The given G^0^ labels were randomly assigned in a way that was consistent with a high degree of predictive structure, but still left the remaining distinctions open to interpretation. We gave participants four labels to choose from to complete the kinship system by labelling the remaining kin. Two of these label choices matched the provided G^0^ labels, and two were novel.^3^ Participants could use as many or as few of the four labels as they liked.

Participants completed eight trials, each with a different underlying kinship system that differently distinguished parents’ siblings.^4^ These can be divided into three types on the basis of distinctions made among parents’ siblings, given in Table 1. Varying the distinctions in the input ensured that our findings about predictive generalisation would be robust across different patterns of surface variation in kin term categorisation.

**Table 1.**
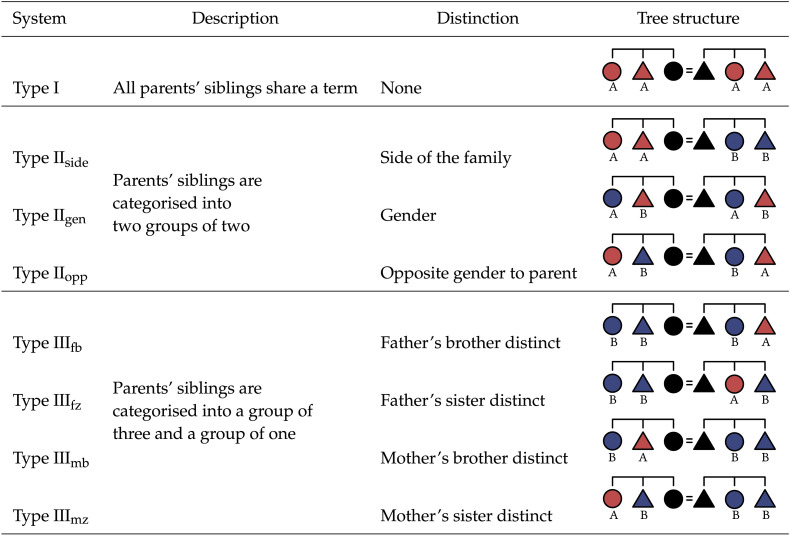
In each trial, participants saw one of eight different kinship systems. Kinship systems varied by which distinctions were present among G^+1^ kin.

We designed eight sets of artificial kinship terms, so that participants would see different kin terms associated with each of the eight system types (see Table 2). These were generated by randomly concatenating syllables from a randomly selected inventory for each of the eight systems. Diacritics were added to each language to further distinguish each set of kin terms. Each set of kin terms was randomly paired with one of the eight system types at the start of the experiment, and the terms were randomly assigned to meanings; no two participants saw the same terms in the same orientation.

**Table 2.** Artificial label sets generated for this experiment. Participants saw different labels in each of their eight trials. Inkeeping with the task framing that they were helping with language documentation in eight under-research languages, each artificial language was given a name. Labels for known kin were randomly chosen from the pool for each language, as were the labels participants could select from.

**Language name**	**Labels**
Zolawȩngï	*kïmy, qïru, bokokohï, zuvewu, mȩwaqïfe, morïky, ryvïri, lobowaby, bybï, hïlaki*
Pukepapu	*kawuka, goviku, lawolu, popingi, wupu, ngapapu, powisunge, lepego, luge, pygele*
Øwar	*aqinudød, øqoç, aweh, orinoçim, iciqusem, iqur, ømenad, adeçim, emøyeç, uquçun*
Aluwut	*azuf, egezalat, ozatof, otev, ulywaf, idanuluf, agivud, ilug, etupol, awupav*
Khêvkkêf	*kvurddåh, rhymsvåd, rhåv, ktot, tsåvvses, svik, ktåvrhus, smêvskêv, vrêd, sfêvdok*
Vpižodvipe	*vbāžo, rbāranžā, nžodo, pžicā, žbodu, vropcāre, vruži, ngcāžo, byure, cžunge*
Tuuquhaïbö	*šiïnö, cöahido, šaaqaude, nuubö, töeqo, tuumïhu, yaöpe, beiguumu, canšuehu, möïtu*
Dăonuu	*taobyo, nyumuo, goozăi, qăo, niuhiê, baumiă, gêă, huanăa, nyumuo, hyonăo*

#### Procedure.

The experiment was implemented with jsPsych (de Leeuw et al., 2023), and ran online in participants’ web browsers.

In the task instructions, participants were told they were helping with a language documentation task, where intuitions about kinship terminology were needed to fill gaps in the vocabulary records of newly documented languages. They were told they would have to use the known terms in each language to infer what the missing terms were likely to be, and that they would have a choice of four terms for each gap.

At the start of the experiment, all participants watched a short tutorial on how to interpret a family tree. Participants were asked to imagine themselves at the centre of the diagram, and characters appeared in a tree structure alongside a prompt describing their relationship in English; e.g., “This is your mother”. After completing the tutorial, participants moved on to the eight critical trials of the experiment, one for each system type. Each critical trial had an **observation phase**, where participants were exposed to the known kin terms for each language, and a **selection phase**, where participants had to choose a kin term for unlabelled referents in the family tree (Figure 11).

In the observation phase, we introduced participants to the known kin terms one at a time. We showed them all the terms for G^+1^ relatives, and two labels for G^0^ relatives. The term would appear underneath a character with a prompt, e.g., “Your mother’s brother is called your **popingi**”. We chose to use English kin terms in the prompt to ensure participants understood that the label was a kin term and not a name.^5^ Each term remained on screen beneath its referent after it was introduced, and participants could progress to the next term by clicking ‘Next’.

Once all the known terms had been presented, the selection phase began. One by one, the unlabelled kin in the tree were highlighted and a prompt would appear, e.g., “In this language, what would you call your mother’s sister’s son?” Participants could then choose from four labels. After their selection, that label would appear beneath the referent, and a new referent would be highlighted. They continued in this way until all kin were labelled, at which point they were asked to confirm their choices to move on to the next trial. Kin were highlighted for selection in a random order; this reduced the risk of any linear ordering effects and encouraged participants to pay attention to which person was being highlighted and how they were related to other individuals in the tree. Participants could undo their responses at any time, allowing them to backtrack on earlier labelling decisions, but they could not change the labels that were given in the observation phase. Once per trial, an already labelled individual would be highlighted and the participant was asked to correctly select their label as an attention check.

### Results

Our prediction was that participants’ generalisations would result in kinship systems with a high degree of predictive structure. We analysed the results from this study in two ways. First, we looked at participants’ generalisation choices—did their choices increase the predictive structure of the kinship systems? Second, we measured the symmetric conditional entropy of participants’ output kinship systems and compared this to a simulated baseline distribution, analogous to the analysis in Study 1. We also conducted some exploratory analysis to investigate which kin are most frequently categorised together, and to test whether participants’ responses were influenced by the distinctions made in their input.

#### Generalisation Gives Rise to Predictive Structure.

**Figure 12.**
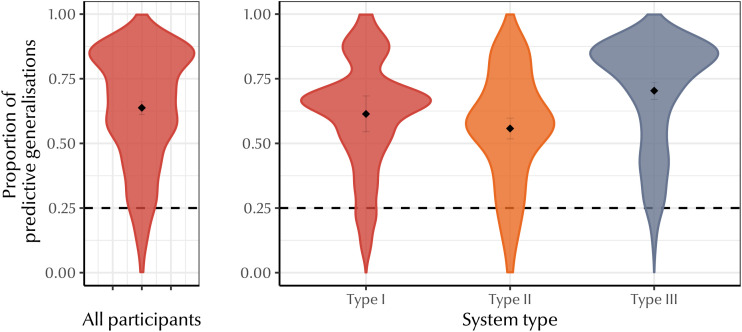
Proportion of predictive generalisations made by participants across all eight trials (left) and grouped by system type (right). Black dots indicate group means, error bars indicate 95% confidence intervals. Dotted line indicates chance—random responses would choose the most predictive generalisation 25% of the time. On the whole, participants make a high proportion of predictive generalisations. However, rarely are their responses 100% predictive—indicating that there may be other factors influencing participants’ generalisations.

For each label a participant assigned to a referent, we coded whether or not their choice led to the greatest possible decrease in symmetric conditional entropy (i.e., greatest increase in predictive structure) among their four options, given the other choices they had made up to that point—1 for the greatest possible decrease, 0 otherwise. This allowed us to measure how many generalisations from known kin terms to a new referent reflected a preference for predictive structure. Figure 12 suggests that across the board, participants often generalised in a way that increased predictive structure. We performed an intercept-only mixed effects logistic regression^6^, predicting predictive generalisations with an intercept and by-participant and by-system random intercepts, revealing that participants generalised predictively significantly more often than we would expect if they did not have a preference for increasing predictive structure (*b* = 0.60, *SE* = 0.15, *p* < 0.001). To confirm whether this effect was robust across system types, we fit a second mixed effects logistic regression, testing whether or not a generalisation was predictive based on a fixed effect of system type (Type I, Type II, and Type III) and by-participant random intercepts and random slopes for system type. Pairwise comparison between levels of the system type fixed effect revealed no significant difference between Type I and Type II systems (*b* = 0.29, *SE* = 0.14, *p* = 0.11), but there was a difference Type I and Type III systems (*b* = −0.38, *SE* = 0.13, *p* = 0.01), and between Type II and Type III systems (*b* = 0.66, *SE* = 0.10, *p* < 0.001). This suggests that the category structure of their input may affect participants’ generalisations.

Participants’ responses were rarely perfectly predictive—only two participants gave 100% predictive responses for any system. But were their responses still more predictive than we would expect given the number of terms they used and how they were distributed? By analogy with the results described in Study 1, we also analysed the symmetric conditional entropy of the kinship systems produced by our participants.

**Figure 13.**
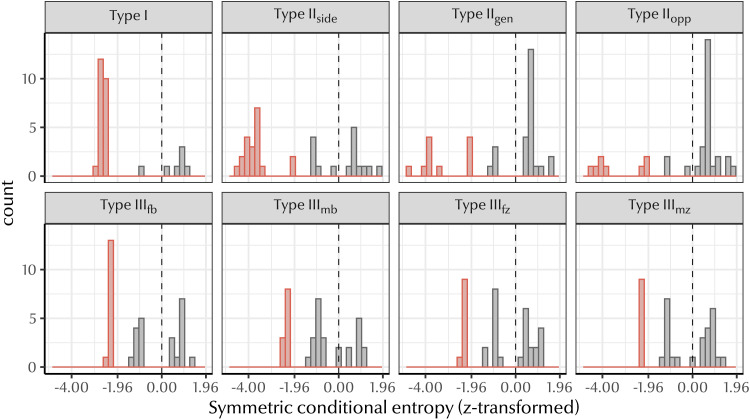
Distribution of symmetric conditional entropy *z*-scores for each system type (see Table 1). Red bars indicate participants who beat a significance threshold of *p* < 0.05. Dashed line shows *z* = 0. Participants produced kinship systems with an extremely high degree of predictive structure for all system types; note that 100% of participants who produced significant symmetric conditional entropy values produced the minimum possible symmetric conditional entropy for that system type. Performance is better for some systems (e.g., Type I) than others (e.g., Type II_gen_ or Type II_opp_), but participants never produce systems with significantly higher symmetric conditional entropy than the baseline.

We expected that participants would produce kinship systems with lower symmetric conditional entropy than chance, indicating that generalising known kin terms to new referents yields kinship systems with greater predictive structure. A lower symmetric conditional entropy in the output language tells us that participants are treating the known terms as informative of the unknown terms—that they are inferring predictive structure. We compared each participants’ responses to a null baseline generated by the same method described in Study 1 for the typological data: each participant’s responses were permuted 1,000 times, randomising which kin terms were associated with which referents. Figure 13 shows each participants’ z-transformed symmetric conditional entropy with respect to their bespoke baseline. Across all eight system types, 37.2% of participant-generated kinship systems have extremely low symmetric conditional entropy compared to their permuted baselines (i.e., *z* < 1.96). This seems a surprisingly small percentage, given that earlier we found that 77.3% of the world’s languages exhibit a significant degree of predictive structure. We attribute this difference to the fact that our experiment permits fewer degrees of freedom compared to the cross-linguistic analysis, and discuss this further below and in Appendix D.

In short, the number of participant-generated kinship systems that have extremely low symmetric conditional entropy supports our hypothesis that generalisation facilitates predictive structure in kinship terminology systems. In the next section, we explore how participants’ preference for predictive structure was modulated by other categorisation preferences.

#### Patterns of Categorisation.

Participants did not always generate kinship systems with a significant degree of predictive structure. So, what kinds of distinctions were participants making when they were not generalising predictively?

**Figure 14.**
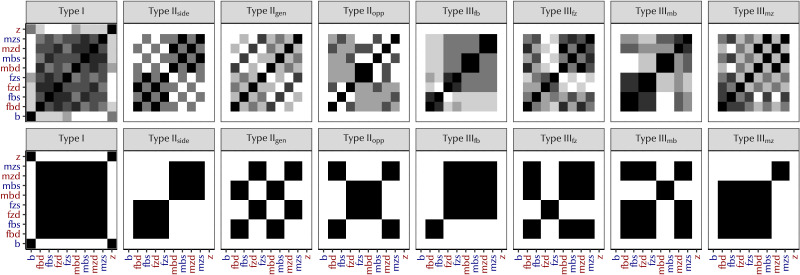
Frequency with which each pair of referents were categorised together for each system type. Top row: Participants’ responses; Bottom row: ideal categorisation given maximally predictive structure (i.e., the lowest possible symmetric conditional entropy). Each cell represents two referents as indicated on the axes, e.g., MZS = mother’s sister’s son, FBD = father’s brother’s daughter. Red axis text indicates female referents, blue axis text indicates male referents. The darker a cell, the greater proportion of participants gave those two referents the same label. Diagonals are black, as each individual is always categorised with themselves. Similarities between the participants’ responses (top row) and the ideal categorisations under our hypothesis (bottom row) indicate closer adherence to predictive structure, while deviations indicate that participants often generalised in a different way. These figures show that participants have a strong tendency to encode predictive structure, but the overlayed checkerboard pattern suggests that a preference to encode gender distinctions is also a strong driver of participant behaviour.

Figure 14 represents the frequency with which referents were categorised together by our participants for each system, compared with the expected frequency assuming only a preference to maximise predictive structure. The greater the resemblance between each pair of plots, the more participants produced predictive kinship systems. For instance, a maximally predictive Type I system is characterised by having all cousins share a term (indicated by the large central square of black cells) and all siblings share a term (indicated by the black corner cells) without any cross-over in sibling and cousin terms (indicated by the white bordering cells). The participants’ responses match this pattern closely, with more participants categorising cousins together than categorising cousins with siblings.

**Figure 15.**
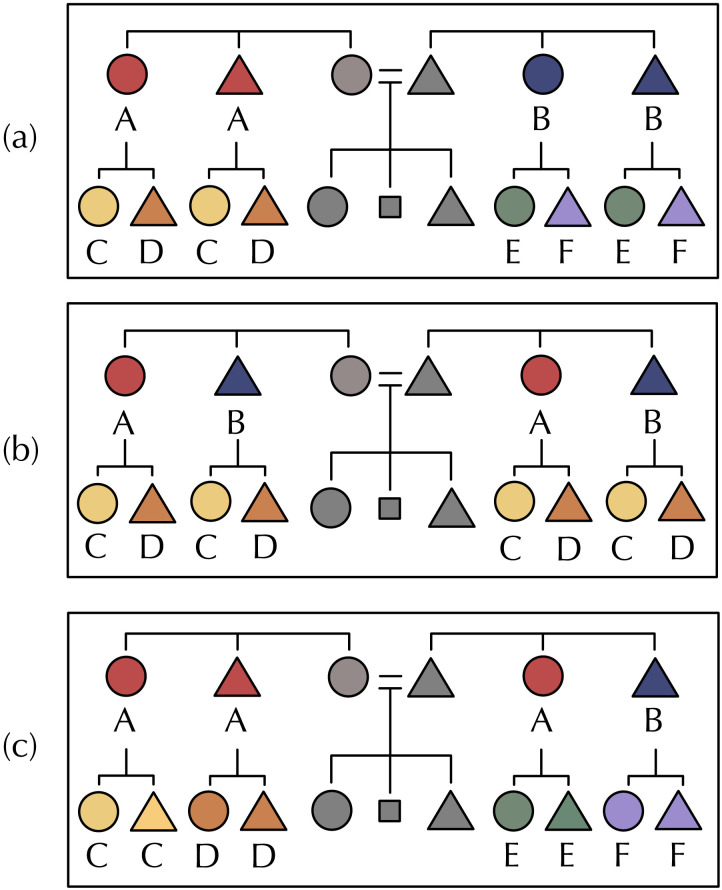
Schematics of participant responses across three system types. Tree conventions as in Figure 2. (a) A predictive Type II_side_ system that also encodes gender among G^0^ kin; while G^0^ terms vary, they vary in a predictable way (e.g., the child of A is always C or D; the child of B is always E or F). (b) A less predictive Type II_gen_ system that only encodes gender among G^0^ kin. Here, children of either A or B can be either C or D; there is no predictive pairing of parents and children. (c) A less predictive Type III_fb_ system that treats each branch of cousins as a discrete unit: children of A are not predictable, as they could be C, D, or E.

Across all systems, we see a strong preference to distinguish referents by gender. Referents are ordered male-female on the axes in Figure 14, so the ‘checkerboard’ appearance of the participants’ responses indicates a preference to categorise male and female referents separately. We can see this behaviour in the plots for Type I and Type II_side_ where the participants’ responses map closely to our expectations with the addition of the checkerboard pattern. This is clearer in some systems than others: for Type II_side_ systems, the checkerboard effect is in high contrast, suggesting that most participants encoded gender distinctions. For Type I systems, it is in low contrast, indicating that participants variably encoded gender distinctions. While encoding gender makes a perfectly predictive system impossible to achieve—since a single parent term will map to two child terms—it is still possible to encode gender in a relatively predictive way. Figure 15a gives an example of a participant response that minimises symmetric conditional entropy to the extent that is possible while still encoding a gender distinction, while Figure 15b exemplifies a non-predictive gender-based system.

Participants’ responses show greater resemblance to the maximally predictive response for some systems more than others: in particular, the responses for Type II_gen_, Type II_opp_ and Type III_mz_ are quite variable. This indicates that participants’ choices were less consistent for these systems. The presence of alternating darker cells suggest that many participants encoded gender distinctions, but the lack of a clear pattern emerging suggests that many participants were swayed away from the most predictive solutions in favour of some other generalisation.

Why might this occur? For Type II_gen_ and Type II_opp_ systems, gender distinctions are encoded among G^+1^ kin. The gender distinctions made in the input for these systems may have encouraged some participants to introduce gender distinctions in G^0^ at the expense of predictive structure. Figure 15b gives an example of a participant response that encodes gender, but does not have particularly strong predictive structure.

However, the distinctions in the input do not always condition participant responses in a straightforward way. For Type III_fb_ systems, father’s brother was distinguished from all other parents’ siblings. While the participant plot for Type III_fb_ looks similar to the ideal plot, we see a pattern of darker cells on the diagonal. This suggests that participants prefer to treat each branch of the tree as discrete—each pair of cousins has a unique term (see Figure 15c)—rather than using the more predictive solution of distinguishing father’s brother’s children from the other cousins.

The categorisation frequencies presented in Figure 14 suggest that the distinctions made in the input affect the distinctions participants will make. Predictive structure is one possible outcome of generalisation, but feature-based solutions like gender or lineality are also available. This can be in addition to predictive structure, as we see clearly for Type I and Type II_side_ systems, or can actually obscure a clear predictive pattern (as we see for Type II_gen_ and Type III_mz_).

#### Influence From Gender Distinctions in Given Labels.

When gender distinctions were present in their input (i.e., for Type II_gen_ and Type II_opp_ systems), our participants’ generalisations were further from our expectations under a preference for predictive structure. We suspected that this reflected a pressure to generalise the feature distinctions present in the given terms—seeing gender encoded in the given G^+1^ terms encouraged participants to encode it in the G^0^ terms.

We coded participants’ responses for whether or not they distinguished terminologically between pairs of gender-distinct referents (e.g., 1 if they distinguished mother’s brother son from mother’s brother’s daughter, 0 if they gave these two the same term). To test whether encoding of gender varied by the distinctions in the input, we fit a mixed effects generalised linear regression model predicting whether or not gender was encoded based on a fixed effect of distinction type in the input (No Distinctions, Gender Distinctions, Non-Gender Distinctions) and by-participant random intercepts and random slopes for distinction type. Pairwise comparison between levels of the model revealed that participants were significantly more likely to encode gender when they saw Gender Distinctions compared to No Distinctions (*b* = 1.59, *SE* = 0.09, *p* < 0.001) or Non-Gender Distinctions (*b* = 1.08, *SE* = 0.07, *p* < 0.001). Participants were also more likely to use gender distinctions when they saw Non-Gender Distinctions compared to No Distinctions, albeit with a smaller effect size (*b* = 0.51, *SE* = 0.08, *p* < 0.001).

However, Figure 14 suggests that our participants show a strong tendency to encode gender distinctions in all systems, regardless of whether gender distinctions are present in the G^+1^ terms or not. What else could be encouraging participants to introduce gender distinctions in this task?

Other than system type (i.e., the observed labels for the generation G^+1^ individuals), the only part of the input that varied were the given G^0^ terms. These were randomly assigned per trial, so the gender of their referents varied between trials. With five possible female referents and five male, it was equally likely a participant saw two labelled G^0^ individuals of the same gender or of different genders in any given trial. When they saw two labelled G^0^ individuals of opposite genders, they may have interpreted this as evidence that the kinship system distinguishes individuals by gender, encouraging them to introduce gender distinctions.

We fit a mixed effects regression with fixed effects for whether or not participants encoded gender and whether or not participants saw gender-distinct labelled G^0^ individuals. This analysis indicated that participants encoded gender more when they saw gender distinctions in the given G^0^ terms (*b* = 0.43, *SE* = 0.05, *p* < 0.001) indicating that participants are swayed towards encoding gender in kin terms when given even a small amount of evidence that the kinship system encodes gender. In the general discussion we consider extra-linguistic reasons why participants might be predisposed to encode gender.

### Discussion

We found that participants are sensitive to predictive structure when generalising kinship terms to novel referents, and often produce kinship systems with a significant degree of predictive structure. These results are consistent with the prevalence of predictive structure in kinship systems that we reported in Study 1, suggesting that this universal may have emerged due to a preference for predictive structure imposed during the generalisation of known kinship terms to new referents.

We recognise that English has a predictive kinship system (*z* = −9.82), so it is possible that our participants may have been influenced by their knowledge of English. However, the results we have presented here do not suggest a strong influence from the English kinship system. Looking at participants’ categorisations in Figure 14, participants do not default to an English-like categorisation that distinguishes only ‘brother’, ‘sister’, and ‘cousin’: darker cells in the corners of the plot for Type I suggest that participants frequently gave the same label to both siblings, (i.e., they did not distinguish them by gender like English) and the ‘checkerboard’ effect we noted in many of these plots suggests a common tendency to encode gender distinctions amongst cousins, which are also not present in English. We also found that participants are influenced by the distinctions in the input language—seeing gender distinctions in the given terms encourages them to use gender distinctions themselves—suggesting that they use the information given to them to complete the task rather than relying on meta knowledge from English.

The degree of predictive structure in our experimental results looks less extreme than in the typological data in Study 1, but it was also harder to beat the baseline in this context. The kinship systems in this experiment had a maximum of 10 terms covering 16 possible meanings, and participants’ choices could only affect the distribution of terms in G^0^. As a result, there were far fewer unique permutations for a given system than for the typological data (see Appendix D for an analysis of the typological data constrained only to these kin types for comparison). In short, predictive structure seems more prevalent in the world than in our experiment, but our results are constrained by the degrees of freedom permitted by our experimental task. In any case, the results are sufficiently extreme as to be unlikely if there were no preference for predictive structure.

However, participants did not always maximise predictive structure; they also have a preference to distinguish kin by features like gender or shared lineage. In our exploratory analysis we found that participants’ responses could vary depending on the distinctions made in their input: gender distinctions amongst labelled G^0^ and G^+1^ kin inspired generalisation of terms to same-gender individuals. While encoding gender distinctions among cousins prevents participants from maximising predictive structure (since the probability of correcting predicting a cousin term given the term for their parent is then only 0.5), our participants generalise as predictively as they can under the extra gender-based constraints they have imposed. Any bias for *maximising* predictive structure can be outweighed in the presence of evidence for a different kind of categorisation, but people still generalise more predictively in these circumstances than we would expect if they had no bias for predictive structure.

## GENERAL DISCUSSION

We set out to identify structural constraints on kin term diversity and the mechanisms that give rise to those constraints, in line with claims that kinship terminology systems are optimised for efficient communication (Kemp & Regier, 2012). Previous work has addressed only one or the other—by suggesting constraints that either cannot explain the entirety of the cross-linguistic variation we can observe or why it emerged (Fox, 1979; Greenberg, 1990; Jones, 2010), or by offering mechanistic explanations drawn from experimental data that use artificial kinship systems removed from what natural language variation looks like (Smith et al., 2020). Here, we have married these two approaches. We conducted the largest-scale typological study of kinship terminology to date and show that systems of kinship terminology near-universally exhibit predictive structure. Following this, we show that participants in the lab have a preference for predictive structure when generalising from known kin terms to new referents. We suggest that the prevalence of predictive structure cross-linguistically can be attributed to this preference.

### Efficiency and Kinship

Our results support the claim that systems of kinship terminology are optimised for efficient communication (Kemp & Regier, 2012). The constraint for predictive structure permits a lot of surface variation in the number of terms and what distinctions they make, while constraining the space of possible systems to those which are predictive. In Kemp and Regier’s (2012) model, simplicity is partly measured by the compressibility of kin term definitions. We argue that predictive structure reflects a pressure for kinship systems to be simple, as kin terms are more compressible under predictive structure: to go back to the example in Figure 1, we can recursively use our definition for *funcle* ‘father and his male sibling’ to generate our definition for *brousin* ‘the child of a *funcle*’. In other words, predictive structure reflects simplicity and is orthogonal to expressivity—kin term meanings are simpler for being predictive, effectively reducing the cost of having more expressive, granular terms.

Simpler, more compressible linguistic structures are associated with ease of learning, while more expressive structures are associated with pressure to encode distinctions (Kirby et al., 2015). We propose here that predictive structure emerges during generalisation: part of the language learning process where some knowledge of the system is available (and learned), but some innovation is still required (Holtz, 2024). When the learner is faced with an inductive problem (like generalising from known terms to new referents who have not yet been assigned a kin term), they do not innovate at random but use their existing knowledge of the system to label the new referent in a systematic way. The internal co-selection process of kin term evolution (Passmore et al., 2021) is facilitated by this process of generalisation: kinship systems evolve to be more generalisable.

### Predictive Structure Beyond Parents and Children

How does our claim about generalisability extend to other kin relationships? Here, we have characterised predictive structure in terms of the similarities between the category structure of kin in G^+1^ and the category structure of their children in G^0^. This approach follows Passmore et al.’s (2021) internal co-selection hypothesis, which refers specifically to the predictiveness between terms for parents and children. However, the metric we have presented here could be extended to test other hypotheses about predictiveness in kinship terminology.

The internal co-selection claim asserts that changes in one part of the system lead to changes in other *related* parts of the system. As such, our metric requires some hypothesis about how parts of a kinship system are related, and why changes in one part of the system might drive changes in another. Here, we have argued that parts of G^+1^ and G^0^ are linked by shared descent. There is a socially-acknowledged connection between parents and their offspring, and we expected that link to be relevant when learners generalise the category structure of their kinship system. There may be other theoretically-motivated connections between kin that would drive a co-selection effect: for instance, there is some evidence that grandparental terms change with grandchild terms in Pama-Nyungan languages (Sheard et al., 2020), a process that could also result in predictive structure.

Indeed, our predictive structure metric could be extended to test whether the category structure of grandparent terms predicts the category structure of grandchild terms (and vice-versa). There is a socially-acknowledged connection between these kin, reflected in many Austronesian languages having a single category for both grandparents and grandchildren (e.g., *vuvu* in Paiwan)—even in English, the morpheme *grand-* is shared across these kin terms. In addition, we can make clear predictions about how the category structures might be similar to each other: both grandparents and grandchildren can be distinguished by features such as gender (men vs women) or gender of the connecting relative (mother’s parents and daughter’s children vs father’s parents and son’s children); these distinctions might be reflected in the category structure of both groups. In this case, symmetric conditional entropy could be calculated on the basis of pairs of individuals with the same feature parameters—e.g., we could test whether having a category of male grandparents predicts a category of male grandchildren and vice-versa.

### Emergence of Feature Distinctions

In our experiment, participants often generalised other properties of the system, such as whether the terms they were given encoded gender distinctions. Our exploratory analysis suggests that gender distinctions in the input increase participants’ willingness to encode gender distinctions amongst unlabelled referents. Elsewhere, feature distinctions emerge without a clear influence from the input. This suggests there may be several biases influencing participants’ behaviour at this task: one that encourages producing kinship systems with predictive structure and one that encourages the repetition of feature distinctions. These are not by definition at odds with one another: participants produce kinship systems with a high degree of predictive structure given the other distinctions they have made.

Innovating distinctions by gender was also common even with only a small amount of evidence that gender was encoded elsewhere in the system. What could encourage this behaviour? Our participants are native English speakers, which could explain their willingness to encode gender distinctions due to the *brother/sister* and *aunt/uncle* distinction in English. However, English does not have a gender distinction for cousins, and yet many of our participants distinguished parents’ siblings children by gender.

There are a number of extralinguistic reasons why our participants may be predisposed to encode gender distinctions so readily. Kin term distinctions in the native language are deeply ingrained to the extent that they modulate performance at cognitive tasks and affect what features of individuals are salient (Anggoro & Gentner, 2003). The characters in our experiment are varied in appearance and look most similar to their immediate relatives, but gender may be the most salient visual characteristic by which to distinguish individuals (at least for our participants). Additionally, models of social discrimination suggest that social categories like gender (and ultimately, gender bias) emerge robustly with minimal pressure to do so (O’Connor, 2019), suggesting that distinguishing individuals by gender may be a readily available hypothesis when faced with tasks like our experiment.

Some participants also introduced a branch distinction, grouping each pair of cousins together as a discrete unit, particularly for Type III systems. Native English speakers are used to bilateral systems of descent, where you perceive yourself to be related equally to your mother and father’s sides of the family. They are more familiar with symmetry rather than lineage as a structuring element of kinship terms: in English, distinctions are symmetrical on each side of the family tree. This may have made our participants reluctant to introduce asymmetrical distinctions (where e.g., mother’s siblings and their children are distinguished at a finer grain than father’s siblings and their children), even where this would be the most predictive solution for Type III systems. It is possible that respondents from unilateral societies (e.g., Hindi speakers, who have additional distinctions between father’s siblings compared to mother’s siblings) might respond differently.

However, culturally-motivated biases and visual cues do not form the entire basis of participants’ behaviour, as predictive structure is not culturally motivated nor visually salient, yet it frequently emerges.

### Kinship Terminology and Social Practice.

We have treated kinship terminology primarily as a linguistic phenomenon, and predictive structure as a universal shaped entirely by biases associated with language transmission. The social dimension of kinship is also relevant to how we communicate about kin. While it is important to point out that measures of communicative efficiency for kinship do take into account that the need to refer to particular kin varies cross-culturally (Kemp & Regier, 2012), the role of culture-specific social structure has largely not been encoded into cognitive models.

Within anthropology, it has long been accepted that patterns of kinship terminology reflect patterns of social structure, and that this is a driving force in kin term variation (Murdock, 1949). For instance, tolerance to cross-cousin marriage may be associated with terminology that distinguishes marriageable kin from non-marriageable kin (Viveiros de Castro, 1998). Recently, with the availability of large cross-linguistic datasets, we see that correlations between kin term organisation and social practice may be confined to particular language families or regions, and may not be as robust as previously thought (Passmore et al., 2023; Passmore & Jordan, 2020). Nonetheless, even if the link between terminology and social practice is not straightforward, future work should consider the interplay between efficient communication and social practice, by taking advantage of the large linguistic and cultural datasets now available to inform cognitive scientific models. Accounts of kin term organisation should acknowledge how kin distinctions are shaped by the repeated imposition of culturally-motivated communicative need, determining how often we communicate about particular individuals, who we perceive to be similar, and what distinctions we need to make.

That said, our experimental design offers a new paradigm for robustly testing claims about kin term diversity that are *removed* from participants’ own cultural frames of reference—to the extent that that is achievable. Artificial language experiments in general allow us to test linguistic claims in a language- and culture-neutral way. In introducing this task framing, we hope to provide a new avenue for testing claims about kinship and cognition, particularly in the light of new contributions to cognitive science that use kinship terminology as a case study to develop models of lexical acquisition and semantic refinement (e.g., Markham et al., 2024; Mollica & Piantadosi, 2022).

## CONCLUSION

We presented a large-scale typological analysis of kinship terminology, showing that the world’s languages exhibit an extreme degree of predictive structure between generations of kin terms. We also conducted an artificial language experiment, finding that participants often generalise known kin terms to new referents in a way that increases predictive structure. We propose that the cross-linguistic tendency for kinship systems to have predictive structure emerges due to pressures imposed during cultural transmission: when individuals are faced with incomplete input when learning kin terms, they will predict the remaining kin terms based on their existing knowledge, resulting in highly structured systems.

## ACKNOWLEDGMENTS

We extend our thanks to our reviewers, Charles Kemp and one anonymous reviewer, for their valuable input on this work.

## FUNDING INFORMATION

MH gratefully acknowledges funding from the Economic and Social Research Council (grant ref. ES/P000681/1).

## AUTHOR CONTRIBUTIONS

MH: Conceptualization; Data curation; Formal analysis; Writing – original draft, Writing – review & editing. FJ: Conceptualization; Writing – review & editing. SK: Conceptualization; Writing – review & editing. KS: Conceptualization; Writing – review & editing.

## DATA AVAILABILITY STATEMENT

All processed data, analyses, and code are available at https://osf.io/4va8k/.

## Notes

^1^ *Uncle* can also denote a parent’s sibling’s husband, or even non-relatives like family friends. Here, we focus only on consanguineal meanings of kin terms—i.e., blood relatives—rather than affinal ones.^2^ It is not uncommon to use orthographic transcriptions of words for wordform similarity analyses in lieu of phonemic transcriptions; the two are reliably correlated (Dautriche et al., 2017).^3^ We gave participants a choice of four labels after finding in a pilot version of the study that participants provided with only two labels commonly used those labels to distinguish gender only. With four labels, participants did not have to choose between competing preferences: it was possible to distinguish gender as well as maximising the predictive structure of the system if they chose to.^4^ The labels assigned to parents’ siblings varied for each system, but parent terms always uniquely distinguished ‘mother’ and ‘father’. This was done partially because it is cross-linguistically common for parental terms to be distinct, and primarily to avoid confusion for our English speaking participants who would not be used to parent terms being polysemous.^5^ We do not expect English kin terms in the prompts to have primed any English-like behaviour. The nature of the task means that using an English-like categorisation is not actually possible: where sibling terms are given, they are not gender-distinct, and due to the given G^0^ labels, cousins must be categorised into at least two groups.^6^ All models reported in this paper were run in R (R Core Team, 2024) using the packages lme4 (Bates et al., 2015) and lmerTest (Kuznetsova et al., 2017).

## Figures and Tables

**Figure A1.**
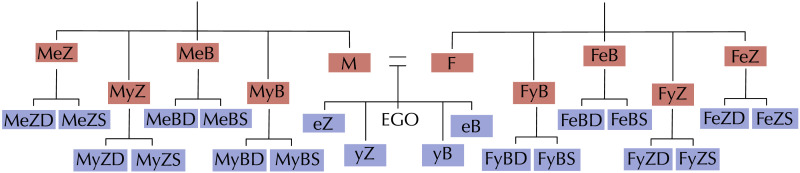
Schematic of the kin types considered in our analysis. Red labels indicate G^+1^ kin, blue labels indicate G^0^ kin. Kin are uniquely identified with shorthand; for example MeZ = mother’s elder sister, FyBS = father’s younger brother’s son. We extracted the terms for these 30 kin types from both the male and female speaker perspective, as these differ in some languages.

**Figure C1.**
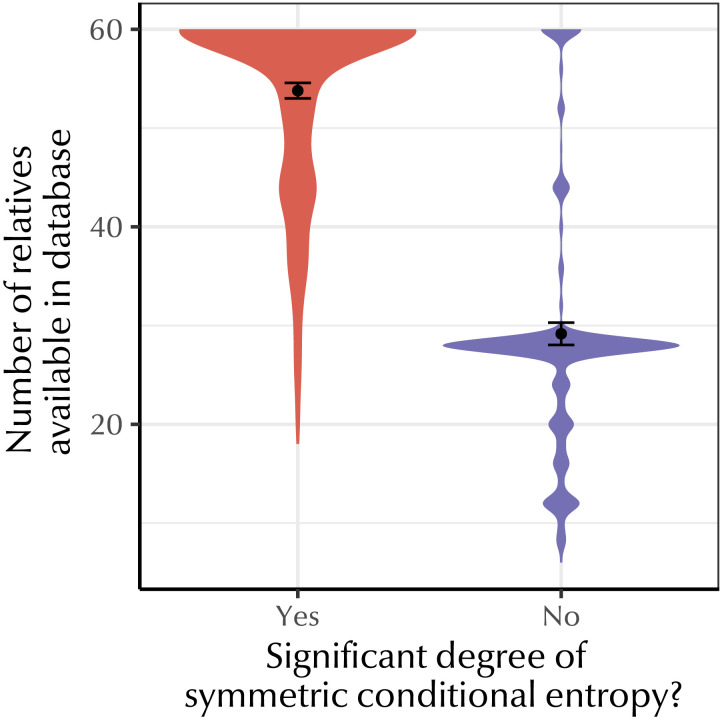
Amount of data available in Kinbank for each language compared against its symmetric conditional entropy significance (against their baseline; significance threshold *p* < 0.05). The maximum number of relatives who could be listed in the database was 60 (across G^0^ and G^+1^). On the whole, languages with non-significant values had less data available, suggesting that the effect size may have been in part an artefact of the data we had available, and that predictive structure may be more prevalent in the real world than we have accounted for in this study.

**Figure D1.**
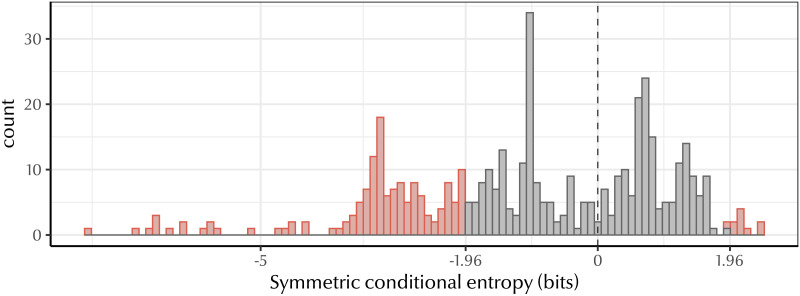
Distribution of *z*-scores for all languages in our dataset when constrained to the 16 kin types used in the experiment. Red bars indicate languages with significant symmetric conditional entropy scores (*p* < 0.05). Dashed line shows *z* = 0. Many languages exhibit a significant degree of predictive structure, though the majority are within our expectations under the null hypothesis that there is no bias for predictive structure. With fewer kin types come fewer possible permutations, making it more difficult for languages to beat the baseline in this version of the analysis.

**Figure E1.**
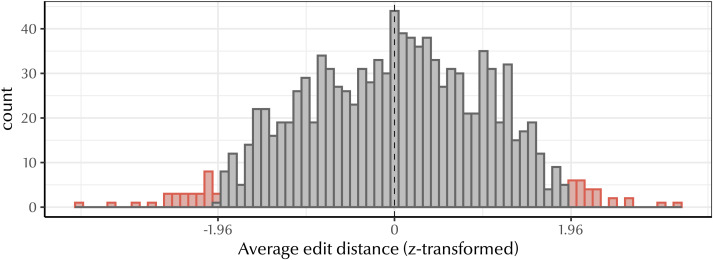
Distribution of average edit distance *z*-scores for all kinship systems in our dataset, but where each term has been replaced with randomly generated strings of the same length. Dotted line gives *z* = 0.

## APPENDIX A: CODING THE KINBANK DATA

Our analysis included 60 kin types across G^0^ and G^+1^, which are given in Figure A1. The Kinbank database makes cross-linguistic comparisons possible by associating each kin term with an objective, language-independent (etic) meaning. For example, in English, the kin term *uncle* is associated with (at least) four etic definitions: ‘mother’s elder brother’, ‘mother’s younger brother’, ‘father’s elder brother’, and ‘father’s younger brother’. These meanings are not distinguished in English kin terms, but they are encoded in other languages—for instance in Myene, which uses the terms *ngw’onèró*, *ngw’erumbe*, *rer’onèró*, and *rer’erumbe* respectively. Regardless of the distinctions made in the terminology of a language, that language will (ideally) have a listing for the full etic grid of kin types in Kinbank.

In cases where more than one term was recorded for a given kin type, we took the first listed term for our analysis, under the assumption that Kinbank lists the more common term first. If two or more alternative spellings were recorded for a kin term, we took the first listed spelling.

Some languages are missing entries in the database. If a language did not have a term listed for one or more kin types, we excluded any pair it was a part of and calculated the symmetric conditional entropy of the remaining terms. For example, if Kinbank was missing a term for MeZ, we would exclude the pairs (MeZ, MeZS) and (MeZ, MeZD) from the analysis for that language. Entire languages were only excluded if the only kin types from Figure A1 that were listed in G_+1_ were M and F; looking only at Ego’s parents and siblings meant that there was no possible variation in predictive structure.

Figure A1 includes a relative age distinction between elder and younger parents’ siblings—we consider the terms for both elder and younger mother’s sister, for example. Rarely, Kinbank only lists a term for MZ ‘mother’s sister’ instead of two separate listings for ‘MeZ’ and ‘MyZ’. In these cases, we did not include this term in the analysis. Our intention was to avoid making assumptions about whether the same term was used for elder and younger siblings in the absence of sufficiently detailed information. Accordingly, we treated the language as if it did not have MeZ or MyZ listed, and calculated the symmetric conditional entropy for any remaining terms.

The reader may notice that the granularity of the etic definitions we used is greater closer to Ego—for example, children of parents’ siblings do not have an age value of their own. Some languages do have a listing for e.g., ‘MeZeD’, but it is much more common in the dataset for age values to be collapsed at this degree. We selected the kin types for analysis which were listed for the most languages, allowing us to include the largest number of languages in our analysis.

## APPENDIX B: A WORKED EXAMPLE OF SYMMETRIC CONDITIONAL ENTROPY

We measured the predictive structure between two generations of a kinship system as the sum of the conditional entropies of each generation of kin terms given the other, a measure we call **symmetric conditional entropy**.

In this Appendix, we will work step-by-step through the calculation of symmetric conditional entropy for the kin terms of Kurdish (see Figure 4a).

In this example, we’ll calculate the conditional entropy of the child terms in G^0^ given the parent terms in G^+1^ first. The conditional entropy of G^0^ given G^+1^ is given by:

$$H\left(G^{0}|G^{+1}\right)=-\sum_{\begin{array}{c} T_{0}\in G^{0},\\ T_{1}\in G^{+1} \end{array}}p\left(T_{0},T_{1}\right)\;{\text{log}}_{2}\;\frac{p\left(T_{0},T_{1}\right)}{p\left(T_{1}\right)}$$ (B1)

where *p*(*T*_0_, *T*_1_) is the probability that *T*_0_ is the label for the child of *T*_1_, and *p*(*T*_1_) is the probability that an individual in G^+1^ has the label *T*_1_.

To calculate this, we need two values: *p*(*T*_1_), the probability of an individual in G^+1^ having the label *T*_1_; and *p*(*T*_0_, *T*_1_), the joint probability of the terms *T*_0_ and *T*_1_ labelling a parent-child pair. For each term in G^+1^, Table B1 gives *p*(*T*_1_).

**Table B1.** Probabilities of terms for G^+1^ kin in Kurdish.

**Kin type**	**Kin term**	*P*(*T*_1_)
MZ	pur	2/6
FZ	pur	
MB	khal	1/6
FB	mam	1/6
M	daik	1/6
F	baok	1/6

There are six kin types in G^+1^ in our Kurdish dataset, but only five kin terms. Each kin term that denotes exactly one kin type has a probability of 1/6, and *pur* has a probability of 2/6 because it denotes two kin types.

For each pair of parent and child terms, Table B2 gives *p*(*T*_0_, *T*_1_).

**Table B2.** Probabilities of each pair of parent-child kin terms in Kurdish.

**Kin type_0_**	**Kin type_1_**	**Kin term_0_**	**Kin term_1_**	*P*(*T*_0_, *T*_1_)
MZ	MZD	pur	purza	4/12
MZ	MZS	pur	purza	
FZ	FZD	pur	purza	
FZ	FZS	pur	purza	
MB	MBD	khal	khaloaza	2/12
MB	MBS	khal	khaloaza	
FB	FBD	mam	amoaza	2/12
FB	FBS	mam	amoaza	
M	Z	daik	khusk	1/12
M	B	daik	bra	1/12
F	Z	baok	khusk	1/12
F	B	baok	bra	1/12

There are 12 unique pairs of parent kin types and child kin types in our Kurdish data. If a pair of terms denotes exactly one pair of kin types, that pair of terms has a probability of 1/12 (e.g., *daik* and *khusk*). If a single pair of terms denotes two pairs of kin types, it has a probability of 2/12 (e.g., *khal* and *khaloaza*). Any pair of terms that do not denote a pair of parent and child kin types has probability 0 (these are not included in the table).

For a pair of terms (*T*_0_, *T*_1_), the conditional entropy *H*(*T*_0_, *T*_1_) is the expected value 𝔼[*f*(*T*_0_, *T*_1_)] where *f*(*T*_0_, *T*_1_) = −*log*_2_$\frac{p\left(T_{0},T_{1}\right)}{p\left(T_{1}\right)}$. Where *T*_0_ = *purza* and *T*_1_ = *pur*:

$$f\left(T_{0}|T_{1}\right)=-{\text{log}}_{2}\;\frac{4/12}{2/6}=0$$ (B2)

which leaves us with a value for each parent-child pair in the Kurdish dataset. The sum of these values is the conditional entropy of G^0^ given G^+1^, given in Table B3.

**Table B3.** Conditional entropies of G^0^ terms given G^+1^ terms in Kurdish.

**H(purza | pur)**	0.000
**H(amoaza | mam)**	0.000
**H(khaloaza | khal)**	0.000
**H(khusk | daik)**	0.083
**H(khusk | baok)**	0.083
**H(bra | daik)**	0.083
**H(bra | baok)**	0.083
**TOTAL** *H*(*G*^0^|*G*^+1^)	0.333

When calculating the conditional entropy of G^+1^ given G^0^, the expected value 𝔼[*f*(*T*_0_, *T*_1_)] where *f*(*T*_0_, *T*_1_) = −*log*_2_$\frac{p\left(T_{0},T_{1}\right)}{p\left(T_{0}\right)}$. The joint probability for a pair of terms (*T*_0_, *T*_1_) is the same as given in Table B4, but this time, the denominator *p*(*T*_0_) is the probability of an individual in **G**^**0**^ being labelled *T*_0_. For each kin term in G^0^, the Table B4 gives *p*(*T*_0_).

**Table B4.** Probabilities of each G^0^ kin term in Kurdish. Note: Z and B each occupy 2/12 of the probability space even though each term is only used for one individual; this is because we count the term for the child of M and the child of F independently.

**Kin type**	**Kin term**	*P*(*T*_0_)
MZS	purza	4/12
MZD	purza	
FZS	purza	
FZD	purza	
MBS	khaloaza	2/12
MBD	khaloaza	
FBS	amoaza	2/12
FBD	amoaza	
Z	khusk	2/12
B	bra	2/12

To illustrate: if *T*_1_ = *pur* and *T*_0_ = *purza*:

$$H\left(T_{1}|T_{0}\right)=-{\text{log}}_{2}\;\frac{4/12}{4/12}=0$$ (B3)

And as before in Table B5, we sum over all conditional entropies for each parent-child pair.

**Table B5.** Conditional entropies of G^+1^ terms given G^0^ terms in Kurdish.

**H(pur | purza)**	0.000
**H(khal | khaloaza)**	0.000
**H(mam | amoaza)**	0.000
**H(daik | khusk)**	0.000
**H(daik | bra)**	0.000
**H(baok | khusk)**	0.167
**H(baok | bra)**	0.167
**TOTAL** *H*(*G*^+1^|*G*^0^)	0.333

And our final symmetric conditional entropy score is the sum of these two values: 0.333 + 0.333 = **0.666**.

## APPENDIX C: ACCESS TO DATA AFFECTS PREDICTIVENESS SCORE

Some languages in our sample had non-significant levels of symmetric conditional entropy. We suggest that, in some cases, this may have to do with the amount of data available for these languages. Kinbank is the most comprehensive database of kinship terminology currently available, and an invaluable resource, but not all languages in Kinbank list complete data for the G^+1^ and G^0^ relatives we look at in this analysis. It is possible that some languages do not encode these meanings at all, or may not place as much importance on them as WEIRD cultures do. Nevertheless, we want to note in this appendix the extent to which variations in the amount of available data had ramifications for our analysis.

We noted that languages with fewer terms are likely to fall within the expected distribution of symmetric conditional entropy values as a result of their having fewer possible permutations, making it harder for the veridical value to surpass the baseline. This may also be the case if a language is missing data on kin types that we expect it to have—the fewer kin terms available for a particular language, the more limited the permutation analysis, and the harder it is for a language to beat its baseline. Figure C1 shows that languages whose predictive structure score was *not* significant (i.e., a *z*-score less than 1.96 and greater than −1.96) tended to have fewer relatives listed in their dataset (the maximum number of relatives we included in our analysis being 60).

Nevertheless, we stress the importance of access to large cross-cultural and cross-linguistic datasets when developing insights on the intersection between language, culture and cognition. Our analysis is particularly susceptible to gaps in this data, making our findings here a conservative estimate of the true distribution of predictive structure cross-linguistically. Irrespective of this, we see it as an enormous advantage that our analysis spans hundreds of languages from all over the globe, a benefit that we are only able to claim thanks to the painstaking work of the researchers collecting data on kinship terminology and those who collated this dataset.

## APPENDIX D: EXPERIMENT RESULTS ARE AS EXTREME AS TYPOLOGICAL RESULTS

We analysed the typological data and our experiment data using Monte Carlo techniques, comparing the true values to a simulated baseline to show that kinship systems (both in the world and produced by our participants) have predictive structure at a greater rate than we would expect if there were no pressure for kinship systems to be predictive.

However, our experiment results were less extreme than the typological results, which we have attributed to reduced degrees of freedom in the experiment. For the typological analysis we included 60 kin types, while the experiment had 16. Reducing the number of kin types greatly reduces the number of possible permutations produced by our baseline, making it harder for the true value to beat the baseline. Here, we show that if our typological analysis were reduced to the same 16 kin types as the experiment, the results would be very similar to our experiment results. It should be noted, however, that the degrees of freedom in the experiment were even fewer, since participants were only able to affect the labels of up to 8 kin types in each trial, as the rest were fixed.

Figure D1 shows the results of this small version of the analysis. While many kinship systems still exhibit a significantly low values of symmetric conditional entropy (29.1%), many more languages are not significant in this version of the analysis (69.3%). These results are similar to the Monte Carlo analysis of the experiment data, where 37.1% of participant-generated kinship systems had an extreme degree of predictive structure.

We do not take this miniature analysis to infer that the results we have presented in this paper are not robust. Rather, we stress that while the experimental results presented in Study 3 may seem less extreme than the typological results presented in Study 1, this is primarily a difference in degrees of freedom. Real kinship systems can vary along many more dimensions than we were able to encode in our experiment design—for example, our experiment does not capture that kin can be distinguished by their relative age, or by the gender of person speaking the kin term. With this miniature version of the typological analysis, we hope to show that the experiment results are about as extreme as the main typological analysis in Study 1, given the reduction in degrees of freedom.

## APPENDIX E: STRING LENGTH DOES NOT IMPACT COMPOSITIONALITY SCORE

In Study 2 we measured the extent to which kin terms exhibit compositionality between pairs of terms for parents and their children. We measured this as the average normalised Levenshtein edit distance between each pair. It is still possible, however, that the length of the kin terms themselves might impact their average edit distance: longer terms are more likely to be morphologically complex than shorter terms, but it is also possible that two longer strings may have more characters in common simply because they have more characters overall.

To confirm that the number of characters in a kin term did not redundantly affect its compositionality score, we recreated the distribution of compositionality scores, but we replaced each kin term in each language with a randomly generated string of the same length as the original kin term. Then we created a null baseline for compositionality using the same method described in Study 2, by permuting which of these random strings was associated with which kin type. This gives us a distribution of compositionality scores for for a dataset where there is definitively no compositionality, only shared string length.

The resulting *z*-scores are normally-distributed around 0, with 95% of languages falling within the significance threshold (*z* > 1.96 or *z* < −1.96; see Figure E1). This is what we would expect from randomly generated data with no bias for compositionality. We compared the distribution of scores for the randomly generated data with the true distribution using a Kolmogorov-Smirnov test, showing that they are significantly different from each other (*D* = 0.257; *p* < 0.001). Given this, we’re confident that the average normalised edit distance we report in Study 2 indeed represents the difference between strings, and cannot be explained solely by the length of the strings themselves.
